# Ixekizumab and complete resolution of enthesitis and dactylitis: integrated analysis of two phase 3 randomized trials in psoriatic arthritis

**DOI:** 10.1186/s13075-019-1831-0

**Published:** 2019-01-29

**Authors:** Dafna D. Gladman, Ana-Maria Orbai, Uta Klitz, James Cheng-Chung Wei, Gaia Gallo, Julie Birt, Suchitrita Rathmann, David Shrom, Helena Marzo-Ortega

**Affiliations:** 1 Division of Rheumatology, Department of Medicine, Centre for Prognosis Studies in The Rheumatic Diseases, University of Toronto, Krembil Research Institute, Toronto Western Hospital, 399 Bathurst St. 1E-410B, Toronto, Ontario M5T 2S8 Canada; 2 Psoriatic Arthritis Program, Division of Rheumatology, Johns Hopkins University School of Medicine, Baltimore, MD USA; 3 Rheumazentrum Ruhrgebiet, Claudiusstr 45, 44649 Herne, Germany; 4 Institute of Medicine, Chinese Medicine Clinical Trial Center, Chung Shan Medical University Hospital, Taichung, Taiwan; 5 Eli Lilly and Company, Indianapolis, IN USA; 6 NIHR Leeds Biomedical Research Centre, Leeds Teaching Hospitals NHS Trust and Leeds Institute of Rheumatic and Musculoskeletal Medicine, University of Leeds, Leeds, UK

**Keywords:** Ixekizumab, Enthesitis, Dactylitis, Psoriatic arthritis

## Abstract

**Background:**

Ixekizumab improves signs/symptoms of psoriatic arthritis (PsA). We present an integrated analysis of baseline disease burden and post-baseline outcomes in ixekizumab-treated patients with enthesitis or dactylitis.

**Methods:**

Data from SPIRIT-P1 and SPIRIT-P2 were integrated. Patients with PsA were randomized to 80-mg ixekizumab every 4 weeks (IXEQ4W) or 2 weeks (IXEQ2W), after a 160-mg starting dose, or to placebo. Inadequate responders at week 16 received rescue therapy. Among patients with baseline enthesitis (Leeds Enthesitis Index [LEI] > 0) or dactylitis (Leeds Dactylitis Index-Basic [LDI-B] > 0), baseline characteristics and disease burden were reported. At week 24, LEI and LDI-B (percentage of patients with resolution [LEI = 0, LDI-B = 0]) were assessed. In pooled treatment groups, the impact of enthesitis or dactylitis resolution on health-related quality of life (HRQoL) (EuroQol-5 Dimensions Visual Analogue Scale [EQ-5D VAS]), physical function (Health Assessment Questionnaire-Disability Index [HAQ-DI]), and pain was assessed.

**Results:**

The integrated analysis set comprised 679 patients; of these, 60% (*n* = 403 of 675) had baseline enthesitis (LEI > 0) and 23% (*n* = 155 of 676) had baseline dactylitis (LDI > 0). At week 24, ixekizumab-treated patients experienced significantly more resolution than placebo of enthesitis (39% IXEQ4W, 35% IXEQ2W, 21% placebo) and dactylitis (78% IXEQ4W, 65% IXEQ2W, 24% placebo). Furthermore, at entheseal points measured by the LEI, ixekizumab-treated patients had significantly higher resolution of enthesitis compared to placebo. At week 24, among all placebo- and ixekizumab-treated patients, resolution of enthesitis was associated with improvements in function and HRQoL whereas dactylitis resolution was associated with more limited improvements. The least squares mean HAQ-DI improvements from baseline were − 0.44 and − 0.25 for patients who did/did not resolve enthesitis, and − 0.41 and − 0.31 for patients who did/did not resolve dactylitis. EQ-5D VAS improvements were 12.3 and 5.8 for patients who did/did not resolve enthesitis, and 10.8 and 9.8 for patients who did/did not resolve dactylitis.

**Conclusions:**

Among patients with pre-existing enthesitis or dactylitis, IXEQ2W- and IXEQ4W-treatment resulted in significant improvements in enthesitis and dactylitis. Enthesitis resolution was associated with improvements in patients’ function, pain, and HRQoL.

**Trial registration:**

ClinicalTrials.gov, NCT01695239, registered on September 25, 2012, and NCT02349295, registered on October 10, 2014.

**Electronic supplementary material:**

The online version of this article (10.1186/s13075-019-1831-0) contains supplementary material, which is available to authorized users.

## Background

Psoriatic arthritis (PsA) is a chronic, inflammatory musculoskeletal disease that frequently affects multiple joints in the peripheral and axial skeleton, with clinical and radiological damage progressing over time [1, 2]. Clinical features of PsA include enthesitis and dactylitis [1], which were highlighted by the Group for Research and Assessment of Psoriasis and Psoriatic Arthritis (GRAPPA) as two of the six commonly accepted clinical domains of PsA that should be considered in treatment decisions [3]. Enthesitis (inflammation at tendon, ligament, or joint capsule’s insertion sites into bone) may be the primary lesion in the development of PsA and was present in 24 to 83% of patients with PsA in various clinical trials using different measures [4, 5]. Similarly, dactylitis (inflammation of the whole digit) is reported to be present in 32 to 48% of patients with PsA [6, 7]; frequently, multiple digits are involved [8]. Real-world evidence suggests that enthesitis and dactylitis are associated with higher disease burden [9].

Biologic therapy for patients with active enthesitis and/or dactylitis is effective and is now recommended for patients who have not responded well to nonsteroidal anti-inflammatories or corticosteroid injections [3, 10]. Ixekizumab is an anti-interleukin-17A (IL-17A) antagonist that has recently been approved for the treatment of active PsA [11–14]. Here, we present a post hoc integrated analysis of SPIRIT-P1 [13] and SPIRIT-P2 [14] to describe the baseline disease burden of enthesitis and dactylitis and outcomes in patients with enthesitis and dactylitis.

## Methods

### Patients and clinical trials

Data were obtained from SPIRIT-P1 (NCT01695239) and SPIRIT-P2 (NCT02349295) [13, 14]. These are randomized, double-blind, placebo-controlled, phase 3 trials involving patients with active PsA previously described [13, 14]. Patients were randomized to subcutaneous injections of placebo, ixekizumab 80 mg once every 2 weeks (IXEQ2W), ixekizumab 80 mg once every 4 weeks (IXEQ4W), or adalimumab 40 mg Q2W (SPIRIT-P1 only; not reported here). Both ixekizumab regimens included a 160-mg starting dose. At week 16, inadequate response (defined by blinded, predefined criteria of < 20% improvement from baseline in both tender joint counts [TJC] and 25 swollen joint counts [SJC]) was required to add or modify concomitant medications. Patients in the placebo group who were inadequate responders at week 16 were given rescue therapy (including conventional disease-modifying antirheumatic drugs [DMARDS], nonsteroidal anti-inflammatory drugs, oral corticosteroids) and were randomized to ixekizumab (IXEQ2W or IXEQ4W) and continued ixekizumab for the remainder of the study. Likewise, inadequate responders who were initially randomized to ixekizumab received rescue therapy and continued with the initial ixekizumab regimen. SPIRIT-P1 and SPIRIT-P2 have similar study designs, with some exceptions; in SPIRIT-P1, patients were biologic-naive, whereas in SPIRIT-P2, patients were experienced with conventional DMARDs and biologic DMARDs.

These trials were compliant with ethical guidelines including the Declaration of Helsinki and other relevant laws and regulations. The protocols were approved by each site’s ethical review committee/institutional review board and all patients provided written informed consent.

### Assessments

Enthesitis was assessed by the presence or absence of tenderness at the 6 sites of the Leeds Enthesitis Index (LEI), lateral epicondyle (left and right), medial femoral condyle (left and right), and Achilles tendon insertion (left and right) [15]. The absence (score = 0) or presence (score = 1) at each of 6 sites is determined; results from the 6 sites are then added to produce an LEI total score ranging from 0 to 6.

Dactylitis was assessed by the Leeds Dactylitis Index-Basic (LDI-B) [16, 17]. Scores based on the circumference and tenderness (presence or absence) of affected digits were calculated and only acute (tender) dactylitis was counted. The results of each digit are then added to produce a total score.

Physical function and health-related quality of life (HRQoL) were assessed by the Health Assessment Questionnaire Disability Index (HAQ-DI) [18, 19] and 5-level EuroQol-5 Dimensions (EQ-5D 5L) instrument, respectively [20]. Patient global assessment (PtGA) [21] and pain visual analogue scale (VAS) were used to assess disease activity and pain intensity, respectively [22]. The HAQ-DI is scored on a scale of 0 (no functional impairment) to 3 (complete impairment) [23]; the minimal clinically important difference (MCID) is estimated to be an improvement from baseline of 0.35 [24]. The PtGA and pain VAS are scored on a scale of 0–100 mm, in which higher scores represent more disease activity or pain intensity, respectively [21, 22]. The EQ-5D comprises a VAS (0–100 scale in which 0 = worst health you can imagine and 100 = best health you can imagine) as well as the EQ-5D descriptive system, which includes 5 dimensions (mobility, self-care, usual activities, pain/discomfort, anxiety/depression) measured in 5 levels ranging from 1 = no problems to 5 = extreme problems. Responses to the EQ-5D dimensions can be used to obtain a single index value, with a range from 1 = full health to 0 = dead [20].

### Statistical analyses

The presence of enthesitis and dactylitis was defined as LEI > 0 and LDI-B > 0, respectively, at baseline. Resolution of enthesitis and dactylitis was defined as LEI = 0 and LDI-B = 0, respectively.

Integrated analyses of the placebo, IXEQ2W, and IXEQ4W groups were performed using data from both SPIRIT-P1 and SPIRIT-P2 trials. An integrated analysis of adalimumab could not be performed because only SPIRIT-P1 had an adalimumab group. Missing data (and data from inadequate responders from week 16 to week 24) were considered as nonresponse for categorical measures (nonresponder imputation [NRI]) or imputed with last observation carried forward (LOCF) method for continuous measures. Comparisons between placebo and ixekizumab treatment groups were performed with a logistic regression model using Wald’s test with treatment and study as factors. In post hoc analyses, associations between HAQ-DI or EQ-5D and enthesitis or dactylitis were based on an analysis of covariance model, adjusting for Disease Activity in PSoriatic Arthritis (DAPSA) change from baseline (LOCF), study, and LDI-B = 0 or LEI = 0 in the model. DAPSA is comprised of SJC + TJC + PtGA + patient pain + C-reactive protein [mg/dL]) [25, 26]. Change from baseline (LOCF) in pain was summarized using mean and standard deviation by patients’ enthesitis/dactylitis resolution status at week 24.

## Results

### Patients and disease burden at baseline

The integrated analysis set comprised 679 patients. At baseline, 60% (*n* = 403 of 675) had enthesitis (LEI > 0) and 23% (*n* = 155 of 676) had dactylitis (LDI-B > 0). Tables 1 and 2 show the baseline characteristics of patients with enthesitis or dactylitis and of all patients within the integrated analysis set.

**Table 1:** Demographic and baseline characteristics (all treatment groups combined)

Characteristic	LEI > 0 (*N* = 403)	LDI-B > 0 (*N* = 155)	Integrated analysis set (*N* = 679)
Age, mean years (SD)^a^	51.2 (11.8)	47.8 (12.1)	51.0 (11.9)
Gender, *n* (%)			
Male	166 (41.2)	70 (45.2)	310 (45.7)
Race, *n* (%)^a^			
White	379 (94.0)	142 (91.6)	629 (92.8)
Black or African American	1 (0.2)	0 (0.0)	3 (0.4)
Asian	16 (4.0)	12 (7.7)	33 (4.9)
Other/multiple	7 (1.7)	1 (0.6)	13 (1.9)
BMI, kg/m^2^, mean (SD)^a^	31.2 (7.8)	29.9 (7.3)	30.2 (7.3)
cDMARD experience, *n* (%)	231 (57.3)	96 (61.9)	385 (56.7)
MTX at baseline, *n* (%)	192 (47.6)	81 (52.3)	318 (46.8)
Time since PsA onset, mean years (SD)	11.0 (8.8)	9.7 (8.0)	11.0 (9.3)
Active PSO with BSA ≥ 3%, *n* (%)^a^	241 (65.7)	105 (74.5)	402 (65.4)
Tender joint count (68 joints), mean (SD)^a^	25.2 (15.5)	22.2 (13.5)	22.0 (14.9)
Swollen joint count (66 joints), mean (SD)^a^	12.5 (9.1)	15.2 (11.3)	11.9 (9.1)
Current enthesitis, *n* (%)^a^	403 (100.0)	108 (69.7)	403 (59.7)
Current dactylitis, *n* (%)^a^	108 (26.8)	155 (100.0)	155 (22.9)
Leeds Enthesitis Index, mean (SD)^a,b^	2.9 (1.6)	3.1 (1.6)	2.9 (1.6)
Leeds Dactylitis Index-Basic, mean (SD)^a,c^	58.2 (72.0)	56.4 (68.3)	56.4 (68.3)

*Abbreviations*: *BSA* body surface area, *BMI* body mass index, *cDMARD* conventional disease-modifying antirheumatic drugs, *LDI-B* Leeds Dactylitis Index-Basic, *LEI* Leeds Enthesitis Index, *MTX* methotrexate, *N* population size, *n* number in group, *PsA* psoriatic arthritis, *PSO* psoriasis, *SD* standard deviation

^a^There are patients with missing baseline information in some groups; the denominator of a particular baseline measure is the number of patients with non-missing baseline measures

^b^Summarized by patients with baseline enthesitis, defined as LEI score > 0

^c^Summarized by patients with baseline dactylitis, defined as LDI-B score > 0

**Table 2:** Disease burden at baseline (all treatment groups combined)

Characteristic, mean (SD)	LEI > 0 (*N* = 403)^a^	LDI-B > 0 (*N* = 155)^a^	Integrated analysis set (*N* = 679)
Pt assessment of joint pain, mm	*n* = 392 63.7 (19.7)	*n* = 151 66.9 (20.7)	*n* = 665 61.4 (21.1)
PtGA of disease activity, mm	*n* = 392 64.6 (20.9)	*n* = 151 68.6 (21.1)	*n* = 665 63.9 (20.8)
HAQ-DI total score	*n* = 391 1.3 (0.6)	*n* = 151 1.3 (0.7)	*n* = 664 1.2 (0.6)
EQ-5D 5L	*n* = 389 0.6 (0.2)	*n* = 151 0.5 (0.2)	*n* = 661 0.6 (0.2)
EQ-5D VAS	*n* = 389 52.5 (19.8)	*n* = 151 52.6 (21.9)	*n* = 661 54.1 (20.9)

*Abbreviations*: *EQ-5D 5L* EuroQoL-5 Dimensions 5 level, *HAQ-DI* Health Assessment Questionnaire-Disability Index, *N* number of patients in analysis population, *n* number of patients with non-missing data, *Pt* patient, *PtGA* patient global assessment, *VAS* visual analogue scale

^a^Baseline enthesitis defined as a baseline LEI score > 0 and baseline dactylitis defined as a baseline LDI-B score > 0

Among patients with baseline enthesitis, the mean (SD) baseline TJC was slightly higher (25.2 ± 15.5) compared to the overall population (22.0 ± 14.9), whereas the baseline SJC was similar to the overall population (12.5 ± 9.1 and 11.9 ± 9.1, respectively). Among patients with baseline dactylitis, baseline TJC was similar to the overall population (22.2 ± 13.5 and 22.0 ± 14.9, respectively) whereas baseline SJC scores were higher than the overall population (15.2 ± 11.3 and 11.9 ± 9.1, respectively); the percentage of patients with a baseline body surface area of psoriasis ≥ 3% was higher than the overall population (74% [*n* = 105 of 141] and 65% [*n* = 402 of 615], respectively) (Table 1). Among patients with either enthesitis or dactylitis, the mean baseline HAQ-DI score was 1.3, which was similar to the burden in the overall population (Table 2).

Among patients with enthesitis (*n* = 389) or dactylitis (*n* = 151) and non-missing data at baseline, approximately 80% reported moderate, severe, or extreme scores for the pain/discomfort domains of the EQ-5D 5L. Likewise, approximately 45 to 51% of patients reported moderate, severe, or extreme scores for the mobility and usual activities domains. (Additional file 1).

### Resolution of enthesitis or dactylitis at week 24

**Fig. 1:**
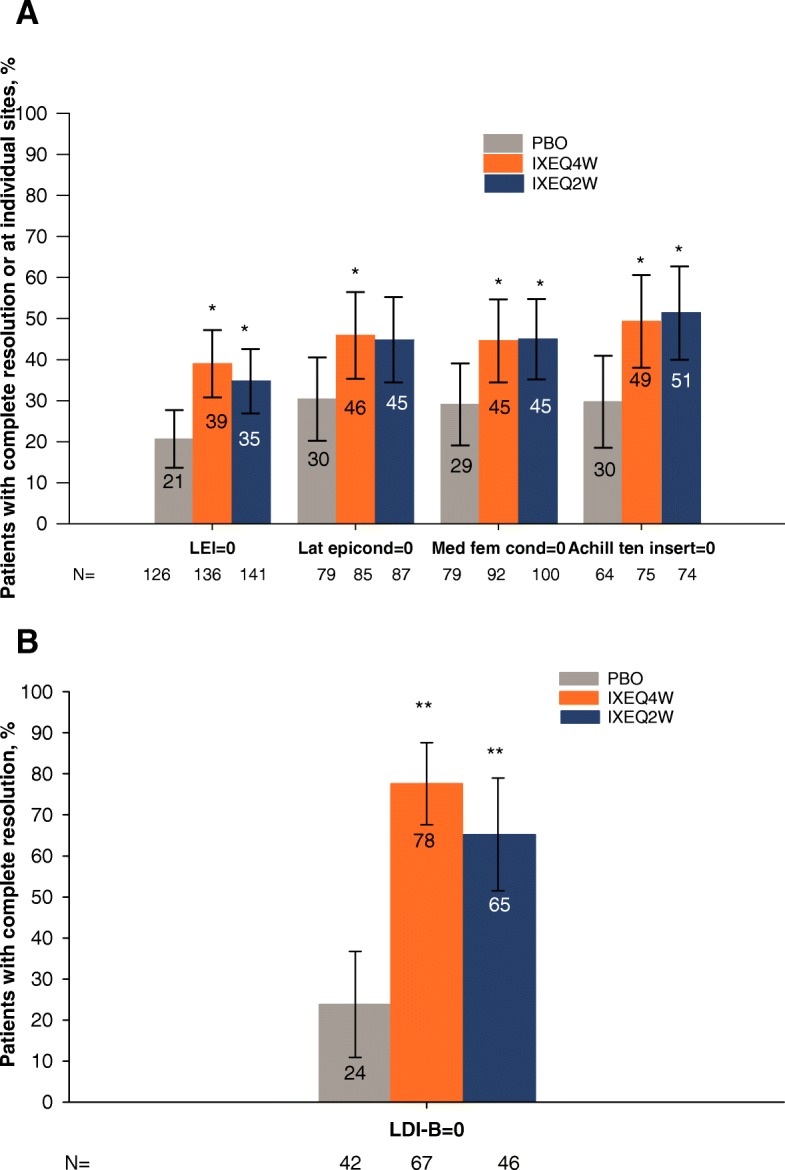
Patients reporting no enthesitis (**a**) and no dactylitis (**b**) at week 24. Integrated analysis set with baseline enthesitis and dactylitis are shown as the percentages of responders (95% CI, NRI). *P vs PBO < 0.05; **P vs PBO < 0.001. *Abbreviations*: Achill tend insert, Achilles tendon insertion; CI, confidence interval; IXEQ2W, ixekizumab 80 mg every 2 weeks; IXEQ4W, ixekizumab 80 mg every 4 weeks; Lat epicond, lateral epicondyle; LEI, Leeds Enthesitis Index; LDI-B, Leeds Dactylitis Index-Basic; Med fem cond, medial femoral condyle; NRI, nonresponder imputation; PBO, placebo

Significantly higher proportions of IXEQ4W (39%)- and IXEQ2W (35%)-treated patients with enthesitis at baseline experienced resolution of enthesitis at week 24 than placebo (21%) (Fig. 1a) in the integrated data set. While results for the adalimumab arm in SPIRIT-P1 were not included in the integrated analyses, 19% (placebo), 33% (adalimumab, *P* = 0.073), 43% (IXEQ4W, *P* = 0.005), and 39% (IXEQ2W, *P* = 0.025) of patients with baseline enthesitis experienced resolution of enthesitis at week 24 [13] in that trial. Likewise, in the integrated dataset, for the lateral epicondyles, medial femoral condyles, and Achilles tendon insertion sites, significantly higher proportions of IXEQ4W-treated patients experienced complete resolution of enthesitis than placebo-treated patients (Fig. 1a). Additional file 2 shows enthesitis resolution of the individual sites of all treatment groups, including adalimumab in SPIRIT-P1.

Significantly higher proportions of IXEQ4W (78%)- and IXEQ2W (65%)-treated patients with dactylitis at baseline experienced resolution of dactylitis at week 24 than placebo (24%) (Fig. 1b). While results for the adalimumab arm in SPIRIT-P1 were not included in the integrated analyses, 25% (placebo), 78% (adalimumab, *P* = 0.001), 80% (IXEQ4W, *P* < 0.001), and 77% (IXEQ2W, *P* < 0.001) of patients with baseline dactylitis experienced resolution of dactylitis at week 24 in that trial [13].

Among patients who did not have dactylitis (LDI = 0) or enthesitis (LEI = 0) at baseline, numerically more placebo patients had a post-baseline LEI > 0 or LDI > 0 than those patients receiving ixekizumab. Among 514 patients with LDI = 0 at baseline, 8/108 (7.4%) patients on placebo had an LDI > 0 at week 24 vs 1/124 (0.8%) on IXEQ4W and 2/142 (1.4%) on IXEQ2W. In the placebo group, 80/180 (44.4%) patients had LDI > 0 at any time post-baseline vs 34/157 (21.7%) on IXEQ4W or 37/177 (20.9%) on IXEQ2W. Among 271 patients with LEI = 0 at baseline, 11/58 (19.0%) on placebo had LEI > 0 at week 24 vs 6/76 (7.9%) on IXEQ4W or 6/69 (8.7%) on IXEQ2W. In the placebo group, 50/97 (51.5%) had an LEI > 0 at any time post-baseline vs 23/93 (24.7%) on IXEQ4W or 18/81 (22.2%) on IXEQ2W.

### Association of resolution of enthesitis or dactylitis with improvement in disability and quality of life

**Fig. 2:**
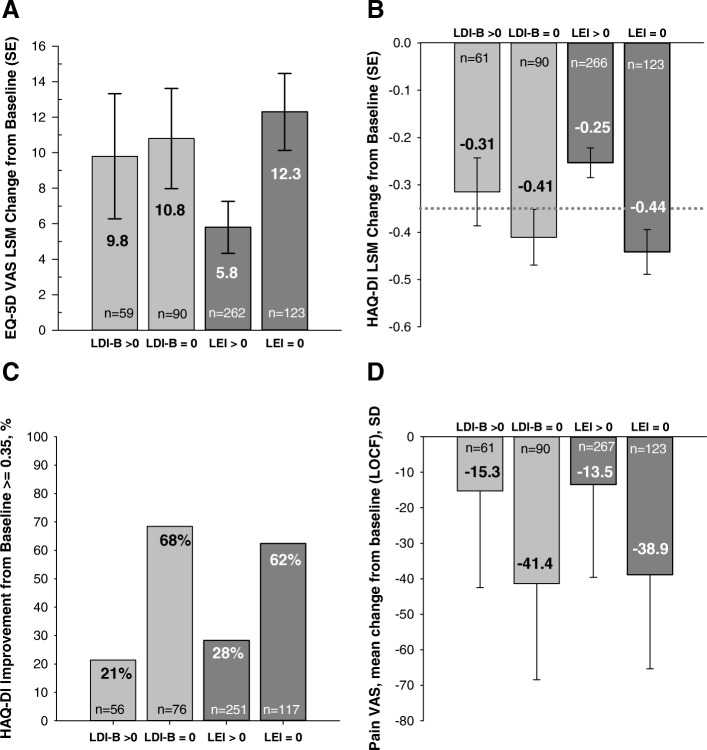
Impact of enthesitis or dactylitis resolution at week 24. Data are from integrated analysis sets of patients with baseline enthesitis and dactylitis. LS mean change from baseline (SE) of EQ-5D VAS (**a**) and HAQ-DI (**b**) scores in patients with enthesitis or dactylitis resolution. **c** Percentages of patients (NRI) with enthesitis or dactylitis resolution reporting improvement meeting or exceeding the MCID of the HAQ-DI (≥ 0.35). **d** Mean change from baseline (LOCF) (SD) of pain VAS. Only one side of the error bar is shown. *Abbreviations*: EQ-5D VAS, EuroQol-5 Dimensions Visual Analogue Scale; HAQ-DI, Health Assessment Questionnaire-Disability Index; LDI-B, Leeds Dactylitis Index-Basic; LEI, Leeds Enthesitis Index; LOCF, last observation carried forward; LSM, least squares mean; MCID, minimal clinically important difference; *N*, population size; *n*, number in group; NRI, nonresponder imputation; SD, standard deviation; SE, standard error; VAS, visual analogue scale

At week 24, resolution of enthesitis symptoms was associated with improvements in patients’ HRQoL (EQ-5D) and function (HAQ-DI), with a larger proportion of patients meeting the MCID for HAQ-DI change from baseline than patients without resolution (Fig. 2a–c). In addition, patients who resolved enthesitis had less pain at week 24 than patients without resolution (Fig. 2d). At week 24, resolution of dactylitis symptoms was associated with a greater proportion of patients meeting MCID in HAQ-DI change from baseline than without resolution, but smaller numerical improvements in patient function and HRQoL than patients resolving/not resolving enthesitis (Fig. 2a–c). Resolution of dactylitis was associated with improvements in patient-reported pain at week 24 (Fig. 2d).

## Discussion

PsA is a heterogeneous disease, with enthesitis and dactylitis as manifestations for many patients. In this cohort, 60% of patients had enthesitis and 23% of patients had dactylitis at baseline, consistent with published estimates of 24 to 83% for enthesitis, but somewhat lower than published estimates of 32 to 48% for dactylitis [5–7]. In this integrated analysis of SPIRIT-P1 and SPIRIT-P2, ixekizumab-treated patients with enthesitis or dactylitis at baseline experienced greater resolution in their enthesitis or dactylitis compared to placebo at week 24. We further found that resolution of these manifestations were associated with improvements in function and HRQoL, irrespective of treatment. Patients who had resolution of their enthesitis/dactylitis symptoms also experienced less pain.

In patients with enthesitis and dactylitis, patient-reported pain and PtGA at baseline were slightly higher than the overall population, whereas HAQ-DI and EQ-5D 5L and VAS were similar to the overall population. Our analyses did not compare baseline disease burden between patients with vs without enthesitis or with vs without dactylitis; however, recent real-world evidence has shown a greater disease burden in patients with, relative to patients without, enthesitis [9, 27, 28]. Using data from the University of Toronto PsA cohort [27], Polachek et al. demonstrated that patients with enthesitis, as measured by the Spondyloarthritis Research Consortium of Canada (SPARCC) enthesitis index, compared to patients without enthesitis had a higher actively inflamed joint count (odds ratio [OR] 1.06, *P* = 0.0002), presence of dactylitis (OR 2.5, *P* = 0.02), presence of tenosynovitis (OR 5.3, *P* < 0.0001), and more pain (OR 1.15, *P* = 0.01). Similarly, in a retrospective, cross-sectional study from the Consortium of Rheumatology Researchers of North America PsA/Spondyloarthritis Registry [9], patients with enthesitis, as measured by the SPARCC, had higher overall work/activity impairment, patient-reported pain, and fatigue compared with patients without enthesitis; however, patients with dactylitis, compared to those without dactylitis, were less impacted based on overall work/activity impairment, pain, and fatigue relative to patients with enthesitis. Differences between real-world evidence and the data presented here may reflect differences in populations enrolled in clinical trials compared to those in the real world.

We did observe that patients whose enthesitis resolved had twice the improvement in quality of life (EQ-5D) compared to patients with residual enthesitis. Pain also improved by more than 2-fold compared to patients who had residual enthesitis symptoms. Greater improvement in functional ability (HAQ-DI) was also seen in patients with resolution of enthesitis vs those with residual entheseal symptoms, and notably more than double the percentage of patients with resolution of enthesitis reported clinically meaningful changes in HAQ-DI compared to patients with unresolved enthesitis. Resolution of dactylitis was associated with smaller improvements relative to enthesitis resolution based on mean change from baseline of HAQ-DI and EQ-5D scores. Similar to the enthesitis analysis, a higher percentage of patients with dactylitis resolution reported clinically meaningful changes in HAQ-DI compared to patients with unresolved dactylitis and percentages of patients with/without dactylitis were similar to those of patients with/without enthesitis; there were also improvements in pain.

The ability to see an association of improvement in physical functioning and quality of life with dactylitis resolution may be limited due to the smaller numbers of patients with dactylitis than enthesitis. Additionally, the model adjusted for DAPSA, which includes a measure of TJC and SJC; the adjustment for joint activity may have confounded analysis of association between dactylitis and functioning/quality of life. The association between dactylitis and HRQoL is inconsistent in literature [9, 28]. Anecdotally, based on personal patient observations made by the authors, patients with dactylitis may not be as symptomatic as patients with enthesitis. Pain was only summarized (i.e., without adjustment for DAPSA) because pain is a component of DAPSA.

Despite the challenges in assessment of enthesitis, we did observe significant treatment effects with ixekizumab for the LEI total score as well as at the individual sites comprising the index (lateral epicondyle, medial femoral condyle, Achilles tendon insertion). Several factors can increase the inter-observer variability of the assessment of enthesitis, such as the identification of the entheseal points, the intensity of the pressure applied, the patient’s pain threshold level, or the presence of other conditions that also cause pain (e.g., fibromyalgia) [5, 29]. Of note, numerically, the largest differences between ixekizumab and placebo in the resolution of enthesitis were observed at the Achilles tendon insertion. This site may be the most reliable site for detecting treatment effects in clinical trials due to the lack of overlap with the tender points of fibromyalgia [30], which can confound its assessment. Additionally, it should be noted that the LEI limits detection to 6 sites, whereas other enthesitis indices measure more sites.

While these results indicate that ixekizumab is effective for the treatment of enthesitis and dactylitis, the SPIRIT studies were not designed to evaluate enthesitis and dactylitis as primary endpoints. Additionally, patients in SPIRIT-P1 and SPIRIT-P2 were not randomized based on enthesitis or dactylitis, enthesitis or dactylitis were not required at entry, nor was a specific symptom threshold specified; inadequate responders were defined based on tender and swollen joint counts. As expected, some patients on placebo who received background medications experienced resolution of dactylitis or enthesitis consistent with published data [27].

Future studies of enthesitis could utilize imaging techniques that can measure pathology such as ultrasound and whole body MRI, which are being investigated [5, 29, 31]. The Outcome Measures in Rheumatology Ultrasound Task Force recently attempted to define enthesitis using ultrasound and included hypoechogenicity, increased thickness of the tendon insertion, calcifications, enthesophytes, erosions, and Doppler activity as core lesions of ultrasound-detected enthesitis, but this work is not fully validated [32]. GRAPPA is also attempting to develop an imaging index for enthesitis [33, 34].

## Conclusions

Treatment with ixekizumab every 2 weeks and every 4 weeks resulted in significant improvements in enthesitis and dactylitis in patients with pre-existing enthesitis or dactylitis. Resolution of enthesitis symptoms was associated with improvements in patients’ function, pain, and HRQoL, irrespective of treatment.

## Additional files

Additional file 1: Patients (%) with enthesitis or dactylitis within the EQ-5D 5L domains at baseline. Patients with with enthesitis or dactylitis are shown. Integrated analysis set with baseline LEI total score > 0 (A). Integrated analysis set with baseline LDI-B total score > 0 (B). The number of patients with non-missing values are 389 of 403 for LEI > 0 and 151 of 155 for LDI-B > 0. (DOCX 89 kb)

Additional file 2: Response rates (%, NRI) for enthesitis by entheseal points at week 24. The intent-to-treat population of SPIRIT-P1 is shown. (DOCX 14 kb)

## Abbreviations

DAPSA: Disease Activity in Psoriatic Arthritis
DMARD: Disease-modifying antirheumatic drug
EQ-5D: 5-Level EuroQol-5 Dimensions version
GRAPPA: Group for Research and Assessment of Psoriasis and Psoriatic Arthritis
HAQ-DI: Health Assessment Questionnaire-Disability Index
HRQoL: Health-related quality of life
IXEQ2W: Ixekizumab 80 mg every 2 weeks
IXEQ4W: Ixekizumab 80 mg every 4 weeks
LDI-B: Leeds Dactylitis Index-Basic
LEI: Leeds Enthesitis Index
LOCF: Last observation carried forward
MCID: Minimal clinically important difference
NSAID: Nonsteroidal anti-inflammatory drug
OR: Odds ratio
PsA: Psoriatic arthritis
PtGA: Patient global assessment
SJC: Swollen joint count
SPARCC: Spondyloarthritis Research Consortium of Canada
TJC: Tender joint count
VAS: Visual analogue scale

## References

[1] Gladman DD, Antoni C, Mease P, Clegg DO, Nash P (2005). Psoriatic arthritis: epidemiology, clinical features, course, and outcome. Ann Rheum Dis.

[2] Ritchlin CT, Colbert RA, Gladman DD (2017). Psoriatic arthritis. New Engl J Med.

[3] Coates LC, Kavanaugh A, Mease PJ, Soriano ER, Laura Acosta-Felquer M, Armstrong AW (2016). Group for Research and Assessment of Psoriasis and Psoriatic Arthritis 2015 treatment recommendations for psoriatic arthritis. Arthritis Rheumatol.

[4] McGonagle D, Conaghan PG, Emery P (1999). Psoriatic arthritis: a unified concept twenty years on. Arthritis Rheum.

[5] Kaeley GS, Eder L, Aydin SZ, Gutierrez M, Bakewell C. Enthesitis: a hallmark of psoriatic arthritis. Semin Arthritis Rheum. 2018. doi: 10.1016/j.semarthrit.2017.12.008 [Epub ahead of print]. 10.1016/j.semarthrit.2018.02.002 29573849

[6] Liu JT, Yeh HM, Liu SY, Chen KT (2014). Psoriatic arthritis: epidemiology, diagnosis, and treatment. World J Orthop.

[7] Brockbank JE, Stein M, Schentag CT, Gladman DD (2005). Dactylitis in psoriatic arthritis: a marker for disease severity?. Ann Rheum Dis.

[8] Ogdie A, Weiss P (2015). The epidemiology of psoriatic arthritis. Rheum Dis Clin N Am.

[9] Mease PJ, Karki C, Palmer JB, Etzel CJ, Kavanaugh A, Ritchlin CT (2017). Clinical characteristics, disease activity, and patient-reported outcomes in psoriatic arthritis patients with dactylitis or enthesitis: results from the Corrona Psoriatic Arthritis/Spondyloarthritis Registry. Arthritis Care Res (Hoboken).

[10] Gossec L, Smolen JS, Ramiro S, de Wit M, Cutolo M, Dougados M (2016). European League Against Rheumatism (EULAR) recommendations for the management of psoriatic arthritis with pharmacological therapies: 2015 update. Ann Rheum Dis.

[11] Eli Lilly and Company. TALTZ-ixekizumab injection, solution [Prescribing Information]. 2018. http://uspl.lilly.com/taltz/taltz.html#pi. Accessed 12 July 2018.

[12] Taltz: EPAR - Product Information. Annex I - summary of product characteristics. 2018. http://www.ema.europa.eu/docs/en_GB/document_library/EPAR_-_Product_Information/human/003943/WC500205804.pdf. Accessed 4 Apr 2018.

[13] Mease PJ, van der Heijde D, Ritchlin CT, Okada M, Cuchacovich RS, Shuler CL, SPIRIT-P1 Study Group (2017). Ixekizumab, an interleukin-17A specific monoclonal antibody, for the treatment of biologic-naive patients with active psoriatic arthritis: results from the 24-week randomised, double-blind, placebo-controlled and active (adalimumab)-controlled period of the phase III trial SPIRIT-P1. Ann Rheum Dis.

[14] Nash P, Kirkham B, Okada M, Rahman P, Combe B, Burmester GR, SPIRIT-P2 study group (2017). Ixekizumab for the treatment of patients with active psoriatic arthritis and an inadequate response to tumour necrosis factor inhibitors: results from the 24-week randomised, double-blind, placebo-controlled period of the SPIRIT-P2 phase 3 trial. Lancet.

[15] Healy PJ, Helliwell PS (2008). Measuring clinical enthesitis in psoriatic arthritis: assessment of existing measures and development of an instrument specific to psoriatic arthritis. Arthritis Rheum.

[16] Healy PJ, Helliwell PS (2007). Measuring dactylitis in clinical trials: which is the best instrument to use?. J Rheumatol.

[17] Helliwell PS, Firth J, Ibrahim GH, Melsom RD, Shah I, Turner DE (2005). Development of an assessment tool for dactylitis in patients with psoriatic arthritis. J Rheumatol.

[18] Fries JF, Spitz P, Kraines RG, Holman HR (1980). Measurement of patient outcome in arthritis. Arthritis Rheum.

[19] Fries JF, Spitz PW, Young DY (1982). The dimensions of health outcomes: the health assessment questionnaire, disability and pain scales. J Rheumatol.

[20] EQ-5D web site. http://www.euroqol.org/. Accessed 18 Jan 2017.

[21] Cauli A, Gladman DD, Mathieu A, Olivieri I, Porru G, Tak PP, GRAPPA 3PPsA Study Group (2011). Patient global assessment in psoriatic arthritis: a multicenter GRAPPA and OMERACT study. J Rheumatol.

[22] Hawker GA, Mian S, Kendzerska T, French M (2011). Measures of adult pain: Visual Analog Scale for Pain (VAS Pain), Numeric Rating Scale for Pain (NRS Pain), McGill Pain Questionnaire (MPQ), Short-Form McGill Pain Questionnaire (SF-MPQ), Chronic Pain Grade Scale (CPGS), Short Form-36 Bodily Pain Scale (SF-36 BPS), and Measure of Intermittent and Constant Osteoarthritis Pain (ICOAP). Arthritis Care Res (Hoboken).

[23] Maska L, Anderson J, Michaud K (2011). Measures of functional status and quality of life in rheumatoid arthritis: Health Assessment Questionnaire Disability Index (HAQ), Modified Health Assessment Questionnaire (MHAQ), Multidimensional Health Assessment Questionnaire (MDHAQ), Health Assessment Questionnaire II (HAQ-II), Improved Health Assessment Questionnaire (Improved HAQ), and Rheumatoid Arthritis Quality of Life (RAQoL). Arthritis Care Res (Hoboken)..

[24] Mease PJ, Woolley JM, Bitman B, Wang BC, Globe DR, Singh A (2011). Minimally important difference of Health Assessment Questionnaire in psoriatic arthritis: relating thresholds of improvement in functional ability to patient-rated importance and satisfaction. J Rheumatol.

[25] Eberl G, Studnicka-Benke A, Hitzelhammer H, Gschnait F, Smolen JS (2000). Development of a disease activity index for the assessment of reactive arthritis (DAREA). Rheumatology (Oxford).

[26] Schoels M (2014). Psoriatic arthritis indices. Clin Ex Rheumatol.

[27] Polachek A, Li S, Chandran V, Gladman DD (2017). Clinical enthesitis in a prospective longitudinal psoriatic arthritis cohort: incidence, prevalence, characteristics, and outcome. Arthritis Care Res (Hoboken)..

[28] Wervers K, Luime JJ, Tchetverikov I, Gerards AH, Kok MR, Appels CWY, et al. Quality of life at baseline in early psoriatic arthritis related to disease domains [abstract 1706]. Arthritis Rheumatol. 2016; 68 (Suppl 10). https://acrabstracts.org/abstract/quality-of-life-at-baseline-in-early-psoriatic-arthritis-related-to-disease-domains/. Accessed 17 Mar 2017.

[29] McGonagle DG, Helliwell P, Veale D (2012). Enthesitis in psoriatic disease. Dermatology.

[30] Marchesoni A, De Marco G, Merashli M, McKenna F, Tinazzi I, Marzo-Ortega H (2018). The problem in differentiation between psoriatic-related polyenthesitis and fibromyalgia. Rheumatology (Oxford).

[31] Orbai AM, Weitz J, Siegel EL, Siebert S, Savage LJ, Aydin SZ, GRAPPA Enthesitis Working Group (2014). Systematic review of treatment effectiveness and outcome measures for enthesitis in psoriatic arthritis. J Rheumatol.

[32] Terslev L, Naredo E, Iagnocco A, Balint PV, Wakefield RJ, Aegerter P, Outcome Measures in Rheumatology Ultrasound Task Force (2014). Defining enthesitis in spondyloarthritis by ultrasound: results of a Delphi process and of a reliability reading exercise. Arthritis Care Res (Hoboken)..

[33] Kaeley GS, D'Agostino MA, Grassi W, Østergaard M, Olivieri I (2012). GRAPPA 2011: proceedings from the ultrasound imaging module. J Rheumatol.

[34] Tom S, Zhong Y, Cook R, Aydin SZ, Kaeley G, Eder L. Development of a preliminary ultrasonographic enthesitis score in psoriatic arthritis - GRAPPA Ultrasound Working Group. J Rheumatol. 2018. 10.3899/jrheum.171465 [Epub ahead of print]. 10.3899/jrheum.171465 30323008

