# Supplementary material for: Ixekizumab and complete resolution of enthesitis and dactylitis: integrated analysis of two phase 3 randomized trials in psoriatic arthritis
Source: Arthritis Res Ther. 2019 Jan 29;21:38. doi: 10.1186/s13075-019-1831-0 (PMC6350390; doi:10.1186/s13075-019-1831-0)

**Additional File 1. Patients (%) with enthesitis or dactylitis within the EQ-5D 5L domains at baseline.** Patients with with enthesitis or dactylitis are shown. Integrated analysis set with baseline LEI total score >0 (A); Integrated analysis set with baseline LDI-B total score >0 (B). The number of patients with non-missing values are 389 of 403 for LEI >0 and 151 of 155 for LDI-B >0.

*Abbreviations*: EQ-5D 5L, EuroQol-5-Dimensions 5 level; LDI-B, Leeds Dactylitis Index-Basic; LEI, Leeds Enthesitis Index.


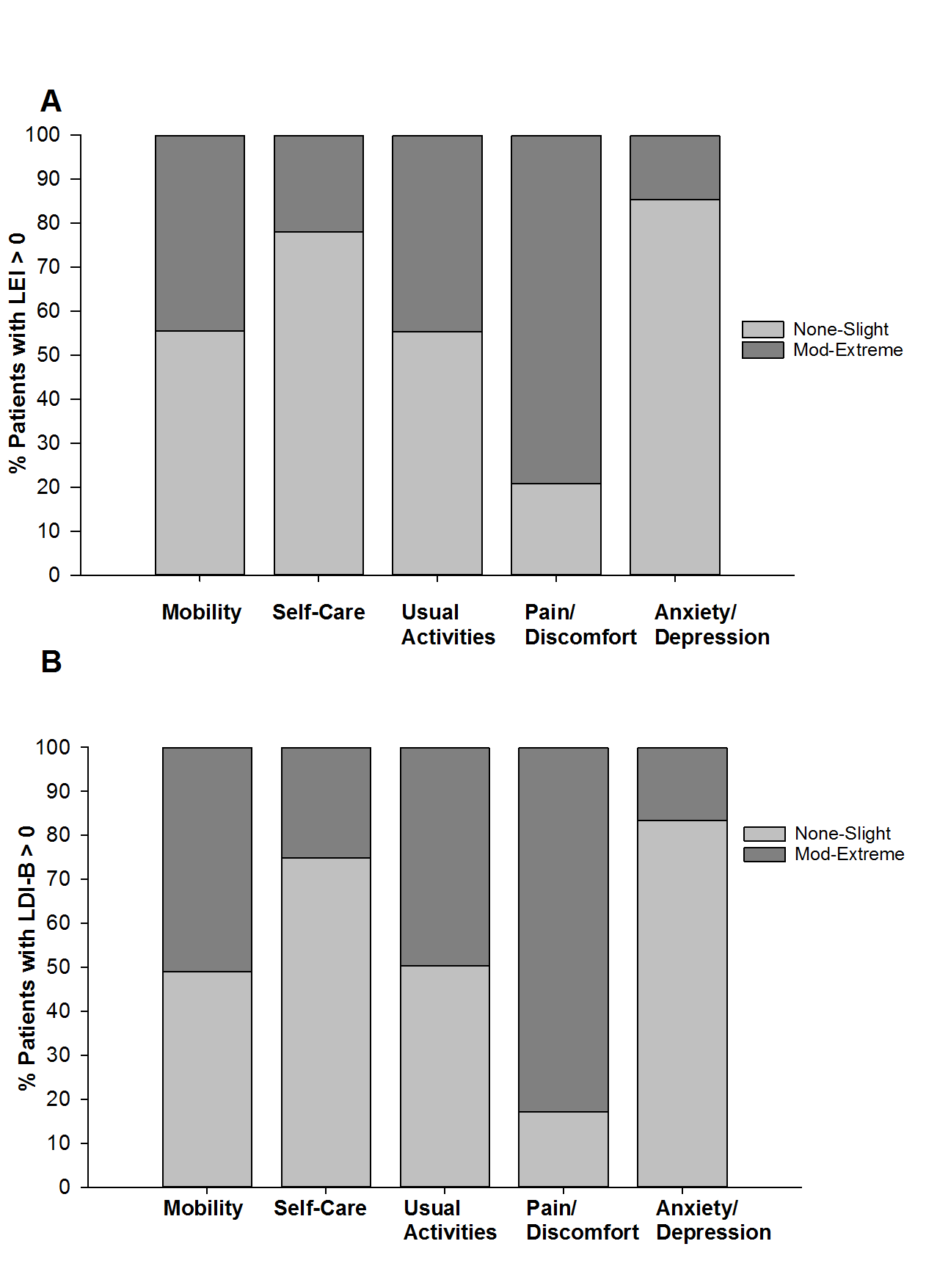

Supplement: Supplementary file 1 — Patients (%) with enthesitis or dactylitis within the EQ-5D 5L domains at baseline. Patients with with enthesitis or dactylitis are shown. Integrated analysis set with baseline LEI total score > 0 (A). Integrated analysis set with baseline LDI-B total score > 0 (B). The number of patients with non-missing values are 389 of 403 for LEI > 0 and 151 of 155 for LDI-B > 0. (DOCX 89 kb) [file 13075_2019_1831_MOESM1_ESM.docx]
