# Supplementary material for: Ixekizumab and complete resolution of enthesitis and dactylitis: integrated analysis of two phase 3 randomized trials in psoriatic arthritis
Source: Arthritis Res Ther. 2019 Jan 29;21:38. doi: 10.1186/s13075-019-1831-0 (PMC6350390; doi:10.1186/s13075-019-1831-0)
Supplement: Supplementary file 2 — Response rates (%, NRI) for enthesitis by entheseal points at week 24. The intent-to-treat population of SPIRIT-P1 is shown. (DOCX 14 kb) [file 13075_2019_1831_MOESM2_ESM.docx]

**Additional File 2. Response Rates (%, NRI) for Enthesitis by Entheseal Points at Week 24. The intent-to-treat population of SPIRIT-P1 is shown**

|  | PBO  (N=57) | ADA  (N=54) | IXEQ4W (N=68) | IXEQ2W (N=57) |
| --- | --- | --- | --- | --- |
| Lateral epicondyle | 10 (27.8%)  n=36 | 16 (43.2%)  n=37 | 21 (45.7%)  n=46 | 17 (42.5%)  n=40 |
| Medial femoral condyle | 9 (23.7%)  n=38 | 12 (32.4%)  n=37 | 22 (48.9%)*  n=45 | 17 (47.2%)*  n=36 |
| Achilles tendon insertion | 7 (23.3%)  n=30 | 14 (48.3%)*  n=29 | 17 (47.2%)*  n=36 | 16 (53.3%)*  n=30 |

* p≤ 0.05

*Abbreviations*: IXEQ2W, ixekizumab 80 mg every 2 weeks; IXEQ4W, ixekizumab 80 mg every 4 weeks; N, population size; n, imputed number; NRI, nonresponder imputation; PBO, placebo.
